# Comprehensive analyses of the occurrence of a fungicide resistance marker and the genetic structure in *Erysiphe necator* populations

**DOI:** 10.1038/s41598-023-41454-1

**Published:** 2023-09-13

**Authors:** Alexandra Pintye, Márk Z. Németh, Orsolya Molnár, Áron N. Horváth, Fruzsina Matolcsi, Veronika Bókony, Zsolt Spitzmüller, Xénia Pálfi, Kálmán Z. Váczy, Gábor M. Kovács

**Affiliations:** 1 Plant Protection Institute, HUN-REN Centre for Agricultural Research, Budapest, Hungary; 2 Department of Plant Anatomy, Institute of Biology, Eötvös Loránd University, Budapest, Hungary; 3 Food and Wine Research Institute, Eszterházy Károly Catholic University, Eger, Hungary

**Keywords:** Fungi, Fungal biology, Fungal genetics, Population genetics, Antimicrobial resistance, Pathogens

## Abstract

Genetically distinct groups of *Erysiphe necator*, the fungus causing grapevine powdery mildew infect grapevine in Europe, yet the processes sustaining stable genetic differences between those groups are less understood. Genotyping of over 2000 field samples from six wine regions in Hungary collected between 2017 and 2019 was conducted to reveal *E. necator* genotypes and their possible differentiation. The demethylase inhibitor (DMI) fungicide resistance marker A495T was detected in all wine regions, in 16% of the samples. Its occurrence differed significantly among wine regions and grape cultivars, and sampling years, but it did not differ between DMI-treated and untreated fields. Multilocus sequence analyses of field samples and 59 in vitro maintained isolates revealed significant genetic differences among populations from distinct wine regions. We identified 14 *E. necator* genotypes, of which eight were previously unknown. In contrast to the previous concept of A and B groups, European *E. necator* populations should be considered genetically more complex. Isolation by geographic distance, growing season, and host variety influence the genetic structuring of *E. necator*, which should be considered both during diagnoses and when effective treatments are planned.

## Introduction

Genetic diversity of plant pathogen populations is affected by several factors, such as reproduction^1^, geographic distance^2^, host genotype^3^ and seasonality^4^. Co-infection, that is, infection of a host by different pathogens^5^ or by different genotypes of the same pathogenic species^6, 7^ can also influence pathogen diversity^5^. Different species of powdery mildew (PM) fungi, belonging to the Erysiphaceae family of the ascomycetes, may co-infect the same host individuals^8–11^, and different genotypes of the same PM species may co-infect the same host^7, 12^. An example is *Erysiphe necator*, the causal agent of grapevine powdery mildew (GPM), as its distinct genotypes co-infect grapevines and cause economically significant epidemics worldwide^13–17^.

*Erysiphe necator* was hypothesized to originate in North America^18^, however, the Asian origin of some genotypes of the species was also assumed^19^. The fungus is introduced to Europe, where two distinct genetic groups, designated as A and B, were identified first based on DNA-fingerprint techniques^20, 21^. Afterwards single nucleotide polymorphisms (SNPs) were assigned to those genotypes, in sequences of the β-tubulin (*TUB2*)^16, 17, 22^, translation elongation factor 1-a (*EF1*-a)^18, 23, 24^ and eburicol 14α-demethylase (*CYP51*) genes^21^, and also in the sequences of the nrDNA internal transcribed spacer (ITS)^21^ and intergenic spacer (IGS) regions^18^.

The biological differences of groups A and B are not well understood. Soon after the discovery of the two *E. necator* groups in European vineyards, studies concluded that these two overwinter in different ways^25, 26^. Group A was reported to overwinter as dormant mycelium within buds^21, 27^. Such infected buds may develop characteristic ‘flag shoots’ to restart the asexual cycle of the fungus in spring^21, 28^. It was also concluded that during winter, group B persists as sexual spores, i.e., ascospores, in chasmothecia, the sexual fruiting bodies of *E. necator*^22, 25, 29^. The proposed association of genetic groups with distinct symptoms, however, was queried by studies reporting that both groups may cause flag shoots^17, 28, 30^.

Some studies found that the reproduction of group A is mainly clonal^21, 29, 31^. However, samplings from different populations showed that sexual reproduction could happen in group A^25, 32^, and, mating-type assays revealed that group A produced chasmothecia and viable ascospores in laboratory conditions^25, 32^. DNA markers of group A in field-collected chasmothecia were also detected, suggesting that sexual reproduction is also possible in natural conditions^16^.

Some studies revealed that group A spread mainly in spring, at the beginning of the season, while group B caused epidemics later in the season^18, 21, 33^. In Hungary, however, group B was detected throughout the vegetation period, and group A was also reported from samples collected later in the growing season, and it was mostly present together with group B^16, 17^. Thus, these results did not support the hypothesis of temporal separation of groups A and B.

The demethylation inhibitor (DMI) fungicides, commonly applied against *E. necator,* may affect the niche partitioning of GPM groups as hypothesized^34^. DMI fungicides (also referred to as azoles) inhibit CYP51, a key enzyme of the fungal sterol biosynthetic pathway, which catalyzes the biosynthesis of ergosterol, a fundamental membrane component of many fungi^35^. The intensive use of DMIs may lead to the spread of fungicide resistance in GPM populations^36^. A marker for DMI resistance is an A to T nucleotide substitution in position 495 (A495T) in the *CYP51* gene of *E. necator*^15, 21, 37^. This mutation results in an amino acid substitution at position 136 (known as Y136F in *E. necator*)^15^. Several studies have shown that the presence of the mutation correlated with high levels of DMI resistance^15, 36–38^. Significant correlation of an other nucleotide substitution, A1119C in *CYP51* with overexpression of the CYP51 enzyme and azole resistance was found in GPM samples from United States and Chile^15, 36^. The correlation of this latter substitution with genetic groups A and B has not been investigated.

In addition to the A and B groups, in Israel a third well-defined group, IL, was found based on microsatellite markers and multi-locus sequencing^19^. The IL group was present from spring to late autumn in the sampled vineyards. Groups A, B^39, 40^ and IL^19^ differed from each other in terms of latency period, the size of lesions caused, and spore production based on laboratory experiments.

It is not understood if temporal succession, differences in fungicide resistance, variability in infection behavior, and other factors such as grapevine cultivars, or co-infection, are responsible for genetic differences between GPM genetic groups. We carried out an intensive sampling to (i) assess the genetic diversity of *E. necator* in Hungarian vineyards, (ii) determine whether there is any genetic differentiation in *E. necator* populations according to season, wine regions, and grape cultivars, (iii) determine the frequency of the DMI-resistance marker A495T, in relation to fungicide treatment, GPM genetic groups and sampling sites; and (iv) investigate the co-infection on single leaves.

## Results

### Fungal samples and genotype diversity

In total, we obtained 7000 sequences and real-time PCR measurements from 2148 GPM DNA samples. The sequences showed variability in *TUB2* nucleotide positions 79 and 368; *EF1*-α positions 33, 336 and 420 and *CYP51* position 495. All ITS and IGS sequences gained from chasmothecia were identical except for three samples differing in ITS position 48 and IGS position 108 (Supplementary Dataset).

**Figure 1:**
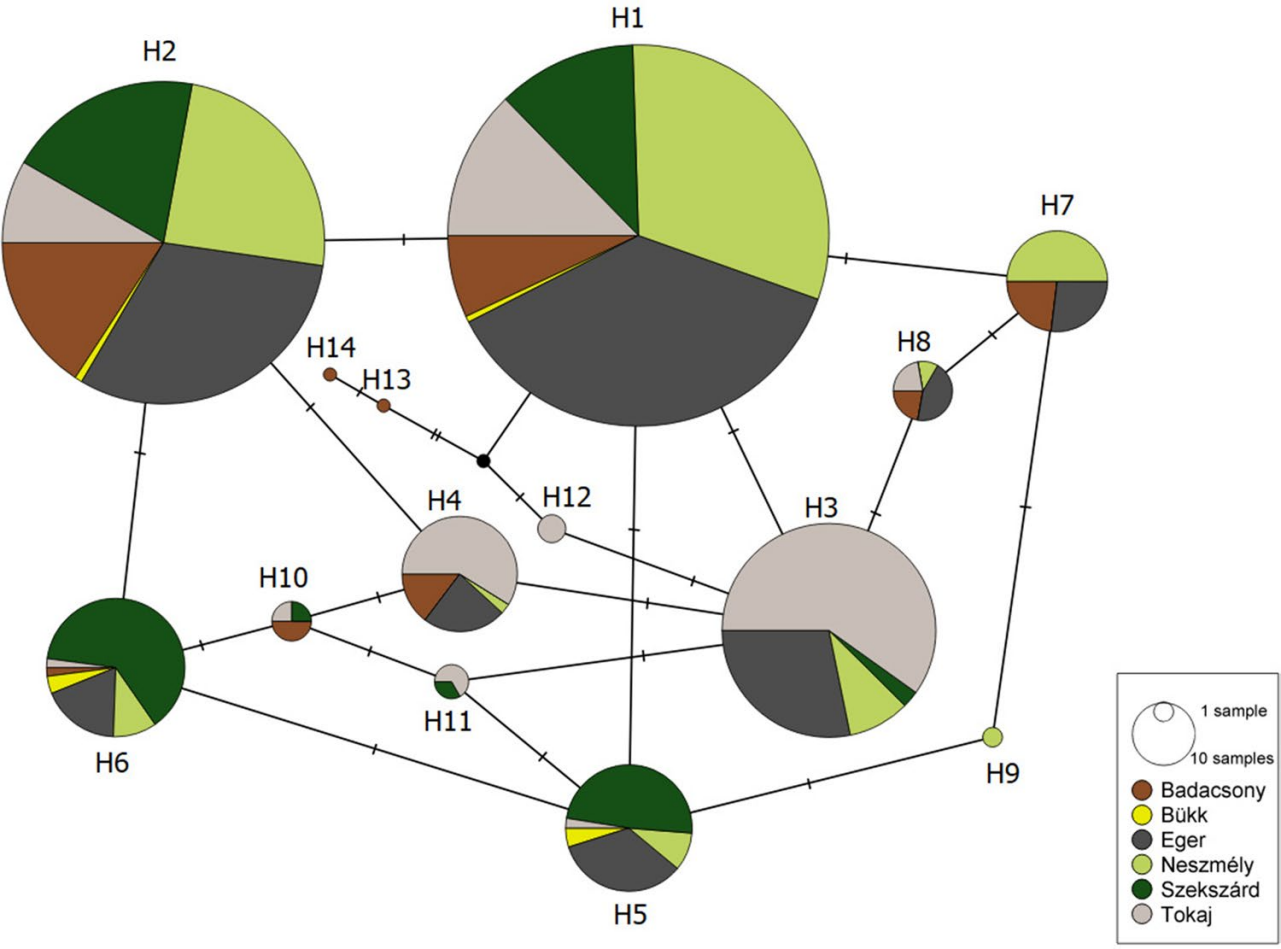
Multilocus haplotype network of genotypes detected in Hungary, based on variable nucleotide positions in partial ITS, IGS, *TUB2* and *EF1-α* sequences. Each circle represents one genotype, and the size of the circle is proportional to the total number of samples (isolates and field samples) belonging to the given genotype. Different colors denote different wine regions, and the size of each circle sector reflects the proportion of samples originating from a given wine region within each genotype. The number of hatches on the branches shows the number of nucleotide differences between genotypes. Genotypes H13 and H14 originate from direct producer grapevines.

Of samples used for defining genotypes (Dataset B), 485 (which include 59 isolates and 426 field samples) had none, while 36 field samples had one ambiguous nucleotide position. We identified altogether 14 genotypes (H1-H14; Table 1). Eight genotypes, H5-6, H8-12 and H14 were detected first in this study. The two dominant genotypes (H1 and H2) had the highest relative frequencies in almost all investigated wine regions (Fig. 1; Table 2). We also identified H5 and H6 in every wine region, except for H5 in Badacsony (Table 2). H7 and H10-H12 were detected only in field samples, but not from isolates.

**Table 1:**
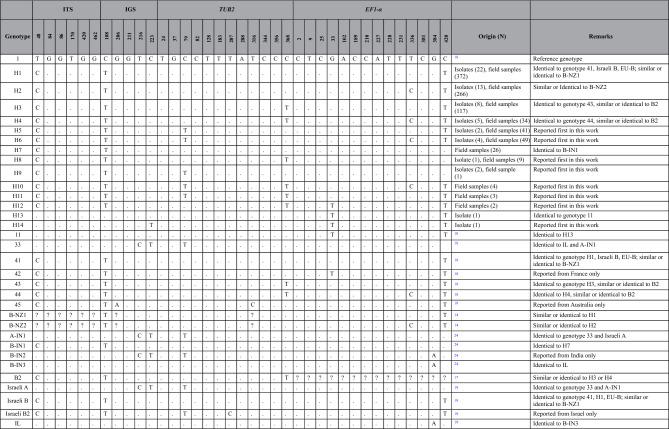
*Erysiphe necator* genotypes based on polymorphic sites in partial sequences of four gene regions.

Hungarian genotypes (H1–H14) are revealed from isolates and field samples of the present study. Genotype 1 was used as the reference genotype^18^. Additional genotypes relevant for comparison detected in other studies are shown. “.” denotes that the nucleotide is the same as in the reference genotype. “?” denotes that the nucleotide was not reported in the study.

**Table 2:**
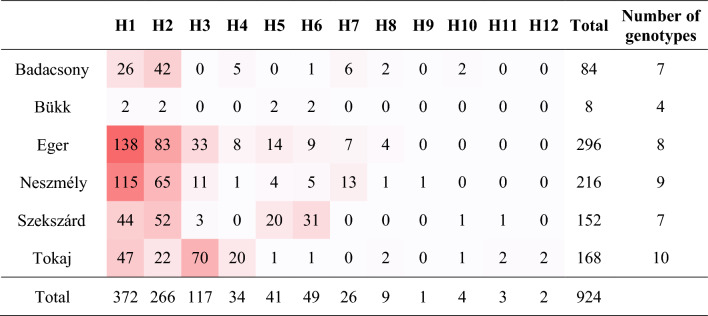
Number of samples belonging to each genotype in the field samples originating from Hungarian wine regions.

Cell color correlates with sample numbers detected in each wine region.

Ten (H1–H6, H8, H9, H13 and H14) of the 14 genotypes were found in the conidial isolates (Table 3). Isolates with two most prevalent genotypes H1 and H2 were found in all sampled wine regions. H1, H2 and H3 were dominant in the early summer and in late autumn. H2 turned up from four, and H3, H5, H6 and H9 from two wine regions. Further genotypes (H4, H8, H9, H13 and H14) were found in less than five isolates, from a single wine region. Genotypes H13 and H14, which differed in a single nucleotide, were detected in isolates from Budapest, originating from two unknown direct producer grape cultivars, but were not detected in field samples. Based on position 79 of *TUB2*, eight isolates belonged to group A (genotypes H5, H6, H9), and these were detected only at the end of the seasons. However, considering *CYP51* sequences, all isolates except for one (from Budapest) belonged to genotype B (Table 3).

**Table 3:** List of isolates obtained in this study, with details on sample origins and *CYP51* genotypes.

Designation	Wine region	Cultivar	Year of collection	Month of collection	Genotype^a^	*CYP51* genotype^b^					
						143	495	608	1119	1170	1620
						(G/T)	(A/T/W)	(T/C)	(A/C)	(G/G)	(C/T)
1A	Budapest	Unknown direct producer hybrid	2018	April	H1	G	A	T	A	G	C
E5K22-2	Eger	Kékfrankos	2018	June	H1	G	A	T	A	G	C
M2F65	Tokaj	Furmint	2018	July	H1	G	A	T	A	G	C
M1H10	Tokaj	Hárslevelű	2018	July	H1	G	A	T	A	G	C
M3F68	Tokaj	Furmint	2018	July	H1	G	A	T	A	G	C
B1	Eger	Blauburger	2019	May	H1	G	A	T	A	G	C
Z1b	Szekszárd	Kékfrankos	2019	May	H2	G	A	T	A	G	C
Z1d	Szekszárd	Kékfrankos	2019	May	H2	G	A	T	A	G	C
EC72	Eger	Chardonnay	2019	July	H2	G	A	T	A	G	C
M2F264	Tokaj	Furmint	2019	July	H1	G	A	T	A	G	C
M2F279	Tokaj	Furmint	2019	July	H2	G	A	T	A	G	C
M2F280	Tokaj	Furmint	2019	July	H3	G	A	T	A	G	C
M3F150	Tokaj	Furmint	2019	July	H3	G	A	T	A	G	C
A	Budapest	Unknown direct producer hybrid	2019	August	H13	T	A	C	A	A	T
3Lb	Eger	Leányka	2019	September	H1	G	A	T	A	G	C
3Lc	Eger	Leányka	2019	September	H2	G	A	T	A	G	C
5La	Eger	Leányka	2019	September	H3	G	A	T	A	G	C
7La	Eger	Leányka	2019	September	H4	G	A	T	A	G	C
7Lb	Eger	Leányka	2019	September	H1	G	A	T	A	G	C
12La	Eger	Leányka	2019	September	H4	G	A	T	A	G	C
15L	Eger	Leányka	2019	September	H2	G	W	T	A	G	C
20L	Eger	Leányka	2019	September	H2	G	A	T	A	G	C
22Lb	Eger	Leányka	2019	September	H5	G	A	T	A	G	C
4Cb	Eger	Chardonnay	2019	September	H1	G	T	T	A	G	C
4Cd	Eger	Chardonnay	2019	September	H2	G	A	T	A	G	C
5Ca	Eger	Chardonnay	2019	September	H1	G	A	T	A	G	C
11C	Eger	Chardonnay	2019	September	H3	G	A	T	A	G	C
2 K	Eger	Tramini	2019	September	H2	G	A	T	A	G	C
7Kc	Eger	Tramini	2019	September	H2	G	A	T	A	G	C
K13	Eger	Tramini	2019	September	H3	G	A	T	A	G	C
11 K	Eger	Tramini	2019	September	H1	G	A	T	A	G	C
13 K	Eger	Tramini	2019	September	H3	G	A	T	A	G	C
4PNa	Eger	Pinot noir	2019	September	H8	G	A	T	A	G	C
6PNa	Eger	Pinot noir	2019	September	H3	G	A	T	A	G	C
6B	Eger	Kékfrankos	2019	September	H4	G	A	T	A	G	C
7B	Eger	Kékfrankos	2019	September	H4	G	A	T	A	G	C
11B	Eger	Kékfrankos	2019	September	H2	G	A	T	A	G	C
5P	Eger	Kékfrankos	2019	September	H3	G	A	T	A	G	C
12P	Eger	Kékfrankos	2019	September	H1	G	A	T	A	G	C
AD	Budapest	Unknown direct producer hybrid	2019	September	H14	G	A	T	A	G	C
SO	Bükk	Olaszrizling	2019	September	H1	G	A	T	A	G	C
5 Da	Badacsony	Kéknyelű	2019	September	H1	G	A	T	A	G	C
10 Da	Badacsony	Kéknyelű	2019	September	H1	G	A	T	A	G	C
11 Da	Badacsony	Kéknyelű	2019	September	H2	G	A	T	A	G	C
S1/6c	Szekszárd	Kékfrankos	2019	September	H1	G	A	T	A	G	C
S2/2a	Szekszárd	Kékfrankos	2019	September	H6	G	W	T	A	G	C
S2/5a	Szekszárd	Kékfrankos	2019	September	H1	G	A	T	A	G	C
S2/11b	Szekszárd	Kékfrankos	2019	September	H6	G	A	T	A	G	C
S4/3	Szekszárd	Kékfrankos	2019	September	H1	G	W	T	A	G	C
S4/4a	Szekszárd	Kékfrankos	2019	September	H2	G	A	T	A	G	C
S2/4b A	Szekszárd	Kékfrankos	2019	September	H9	G	W	T	A	G	C
S2/4b C	Szekszárd	Kékfrankos	2019	September	H6	G	T	T	A	G	C
M3/1a	Tokaj	Furmint	2019	October	H9	G	A	T	A	G	C
M3/1b A	Tokaj	Furmint	2019	October	H5	G	A	T	A	G	C
M3/5	Tokaj	Furmint	2019	October	H3	G	A	T	A	G	C
M4/2b	Tokaj	Furmint	2019	October	H1	G	A	T	A	G	C
N2/2a	Neszmély	Savignon Blanc	2019	October	H1	G	T	T	A	G	C
N2/2b	Neszmély	Savignon Blanc	2019	October	H6	G	A	T	A	G	C
N2/3a	Neszmély	Savignon Blanc	2019	November	H1	G	A	T	A	G	C

^a^Details of each genotype are shown in Table 1.

^b^Only polymorphic sites are listed. Nucleotide positions correspond to the nucleotide positions of the reference GenBank accession U83840.

**Figure 2:**
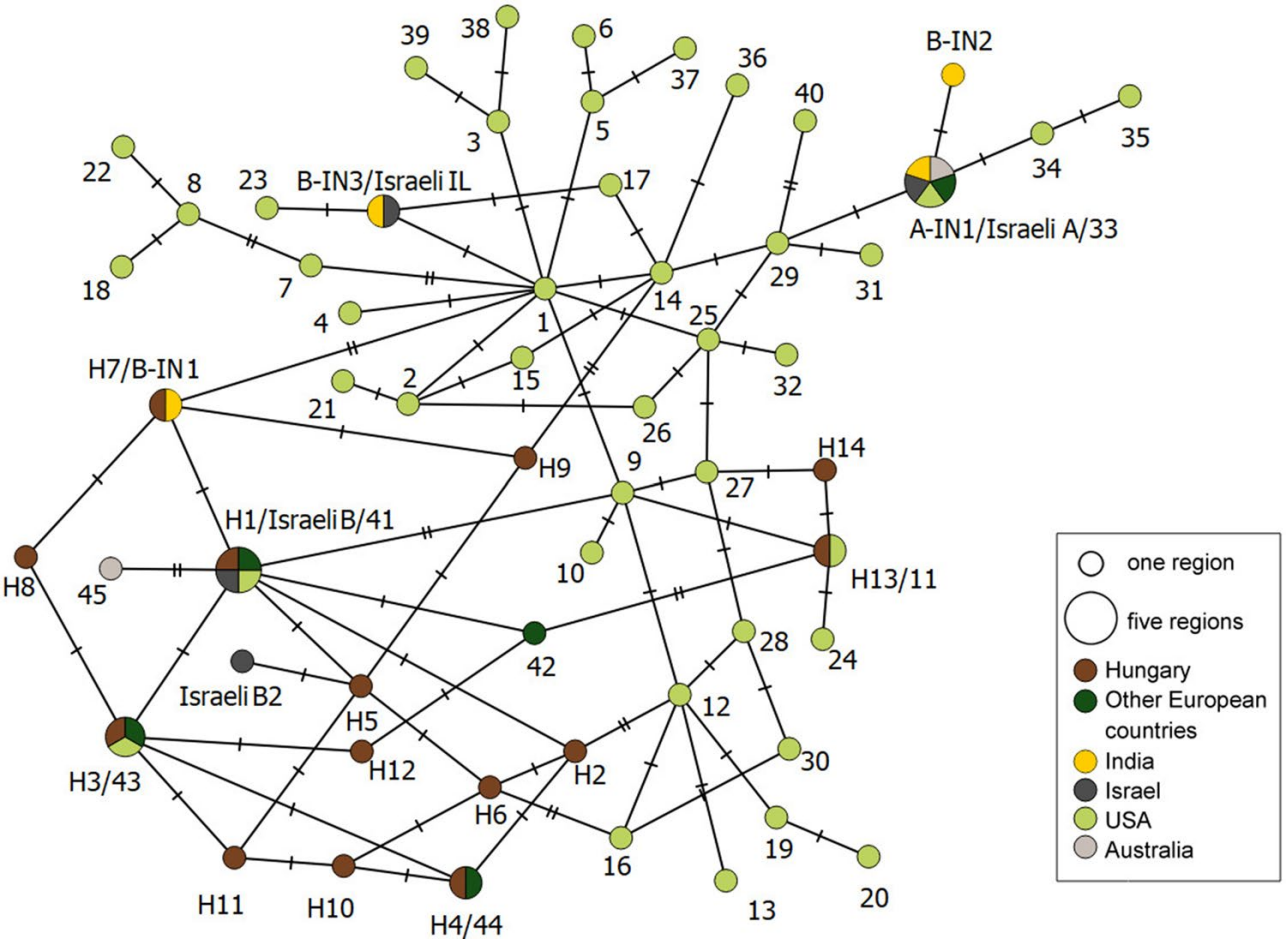
Multilocus haplotype network of genotypes detected in Hungary, other European countries, India, Israel, USA, and Australia, based on variable nucleotide positions in partial ITS, IGS, *TUB2* and *EF1-α* sequences. Each circle represents one genotype, and the size of the circle is proportional to the number of regions where the given genotype was detected. Different colors denote different regions. The number of hatches on the branches shows the number of nucleotide differences between genotypes.

The haplotype network analysis (Fig. 2) showed that the highest number of genotypes was detected in the USA, while the second highest in Hungary. Eight genotypes were solely detected in Hungary. Genotypes from Hungary and the USA were interconnected for a lesser degree. Israeli B and Israeli B2 clustered close to Hungarian samples, while Israeli IL and Israeli A were more similar to the samples from the USA and India. Samples from India were mostly distinct from Hungarian samples, except for one genotype occurring in both locations. Indian samples clustered closer to the genotypes detected in the USA than to the ones from Hungary. Genotype A-IN1 (corresponding to Israeli A, and genotype 33; see below) was the only one detected in five locations, but not in Hungary. None of the genotypes was detected in all the six locations.

### Linkage disequilibrium, population structure and genetic differentiation

**Figure 3:**
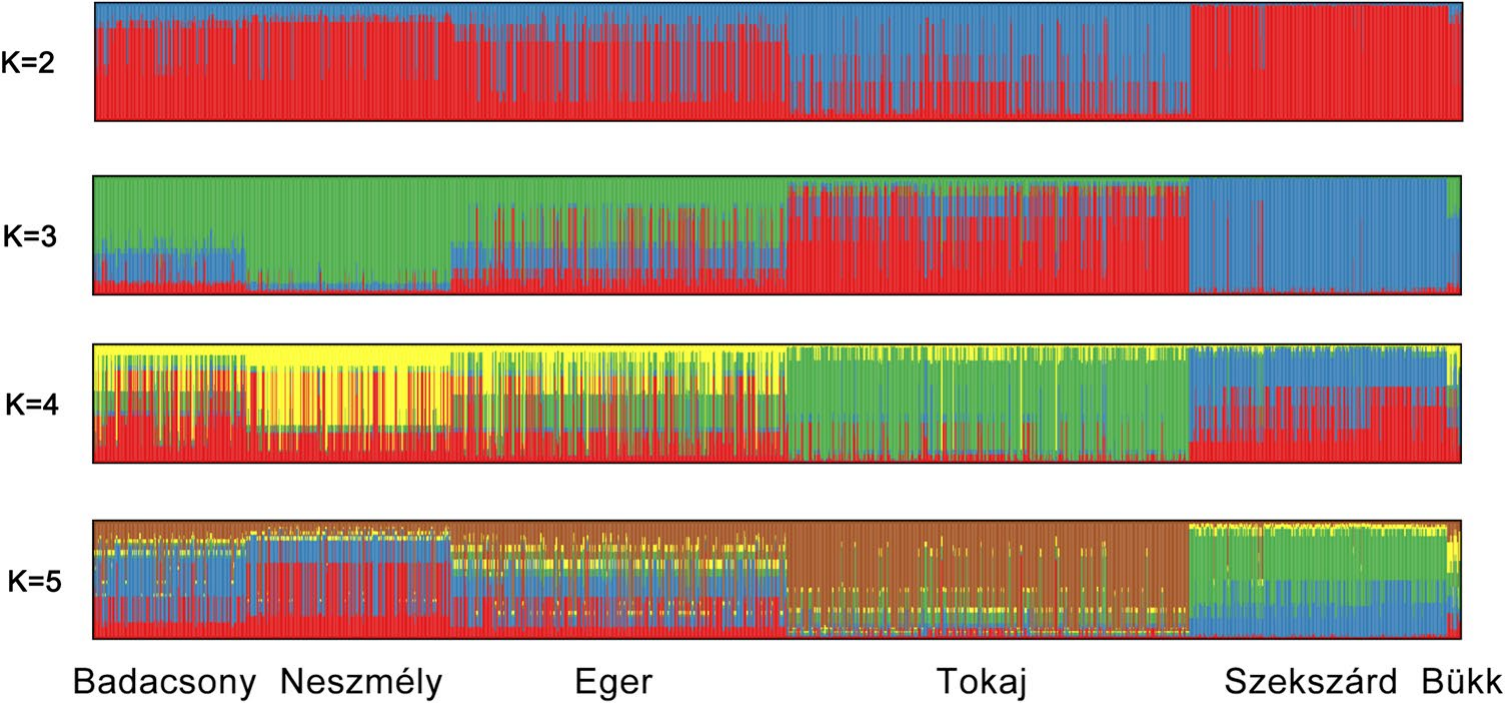
Genetic clustering of *E. necator* samples based on the analysis by STRUCTURE software, for K = 2–5. The geographical origin of strains is indicated on the K = 5 barplot.

Analysis with Multilocus did not detect significant deviance from linkage disequilibrium, the association indexes (I_A_, and $\overline{r }$_d_) did not deviate significantly from zero (*p* = 0.69). The analysis of the population structure of GPM with STRUCTURE yielded well-defined clusters at K values up to 5 (Fig. 3). STRUCTURE Harvester identified K = 5 corresponding to the strongest population structure.

According to genetic differentiation analyzes, there were significant (*p* = 0.001) genetic differences among the populations which were distinguished based on results of STRUCTURE (Table 4). Eger and Neszmély populations were the most similar (PhiPT = 0.013), while Szekszárd and Tokaj were the most different (PhiPT = 0.268). When analyzed as distinct populations corresponding to wine regions, all (*p* ≥ 0.179, except for Szekszárd, *p* = 0.08) were in linkage equilibrium.

**Table 4:** Level of genetic differentiation between Hungarian *E. necator* populations, as calculated by GenAlEx.

	Badacsony	Neszmély	Eger	Tokaj	Szekszárd
Badacsony		0.001	0.001	0.001	0.001
Neszmély	0.025		0.001	0.001	0.001
Eger	0.019	0.013		0.001	0.001
Tokaj	0.131	0.126	0.070		0.001
Szekszárd	0.079	0.142	0.117	0.268	

PhiPT values are shown below the diagonal, *p*-values (based on 999 permutations) are shown above the diagonal.

The genetic diversity of populations was in the range from 0.565 to 0.683, with the lowest being in Neszmély and the highest in Badacsony (Supplementary Table 1). We found that pairwise genetic dissimilarity between populations increased with geographical distance (Mantel test, *p* = 0.01; R = 0.589).

The genetic composition of samples collected in 2017 differed significantly (*p* = 0.001) from those of collected in 2018 and 2019, with the measure of differentiation (PhiPT) being ≤ 0.014. Samples from 2018 and 2019 did not show significant population differentiation (*p* = 0.29). Genetic composition differed significantly between summer and autumn samples (*p* = 0.001) with a PhiPT of 0.129, and also between cultivars Kékfrankos and Chardonnay (*p* = 0.001), with a PhiPT of 0.024.

### Presence of groups A and B in chasmothecia and mycelial samples

Based on nucleotide C or T present in position 79 in *TUB2*, group B was detected in 1017 out of 1099 chasmothecia, while group A was present in 218 chasmothecia. Specifically, only group B was present in 881 (80%), and only group A was present in 82 (~ 7.5%) chasmothecia. Group A and group B were present together in 136 chasmothecia (~ 12.5%). Thus, group A was mostly present together with group B in chasmothecia: in 62% of chasmothecia (136 out of 218) in which group A was detected, group B was also present. Among 311 mycelial samples, group B was present in 282 and group A in 53 samples, including 24 in which both groups were found. The Chi-squared test did not show a significant correlation of the chasmothecial origin of a given sample and its assignation to either genetic group (*p* = 0.338).

### SNPs in *CYP51* associated with DMI resistance

**Figure 4:**
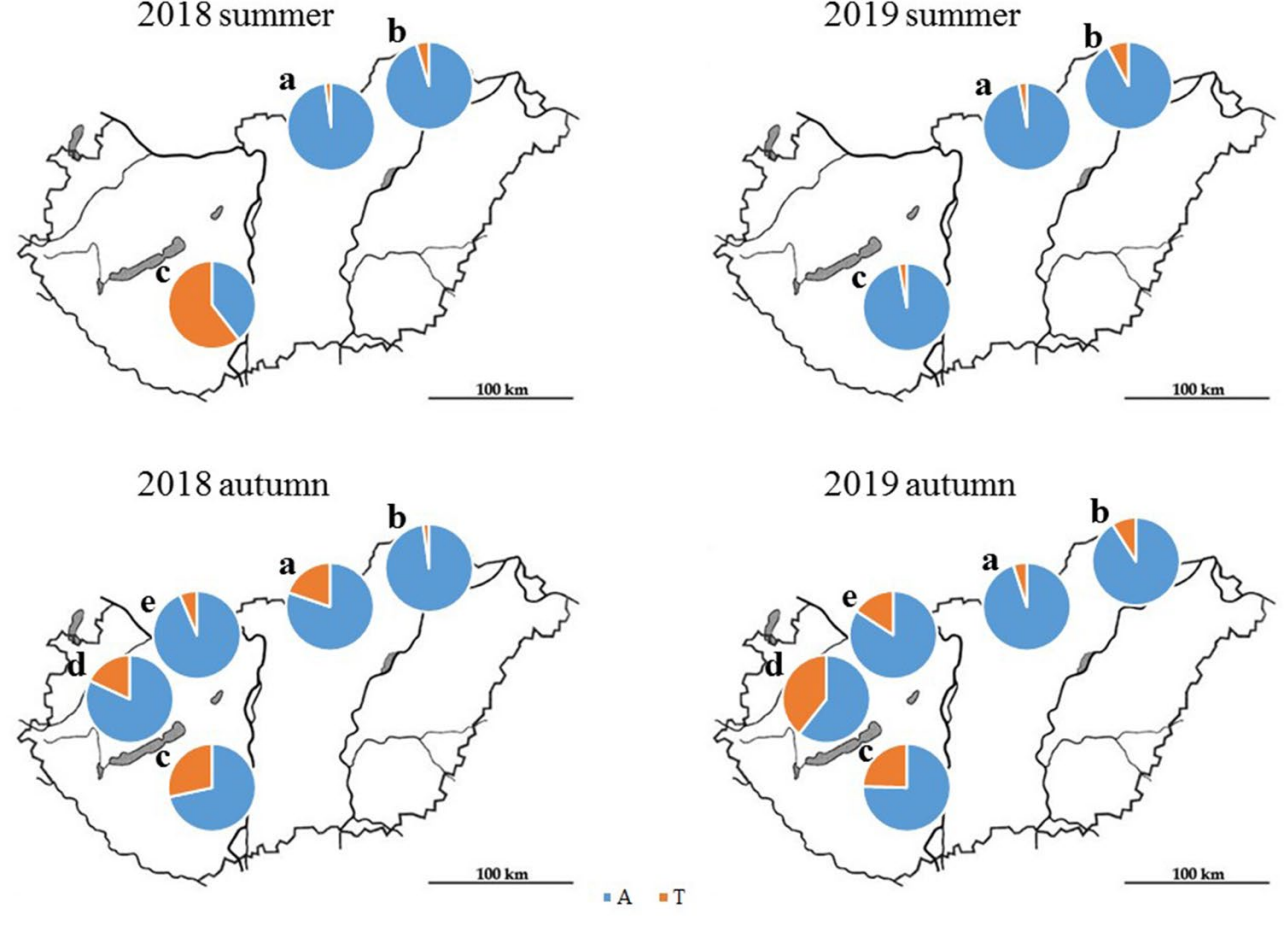
Occurrence of the fungicide resistance marker A495T (shown in orange) in two seasons in 2018 and 2019 in different wine regions. Blue color shows the wild type allele. (**a**): Eger, (**b**) Tokaj, (**c**) Szekszárd, (**d**) Badacsony, (**e**) Neszmély wine regions.

**Figure 5:**
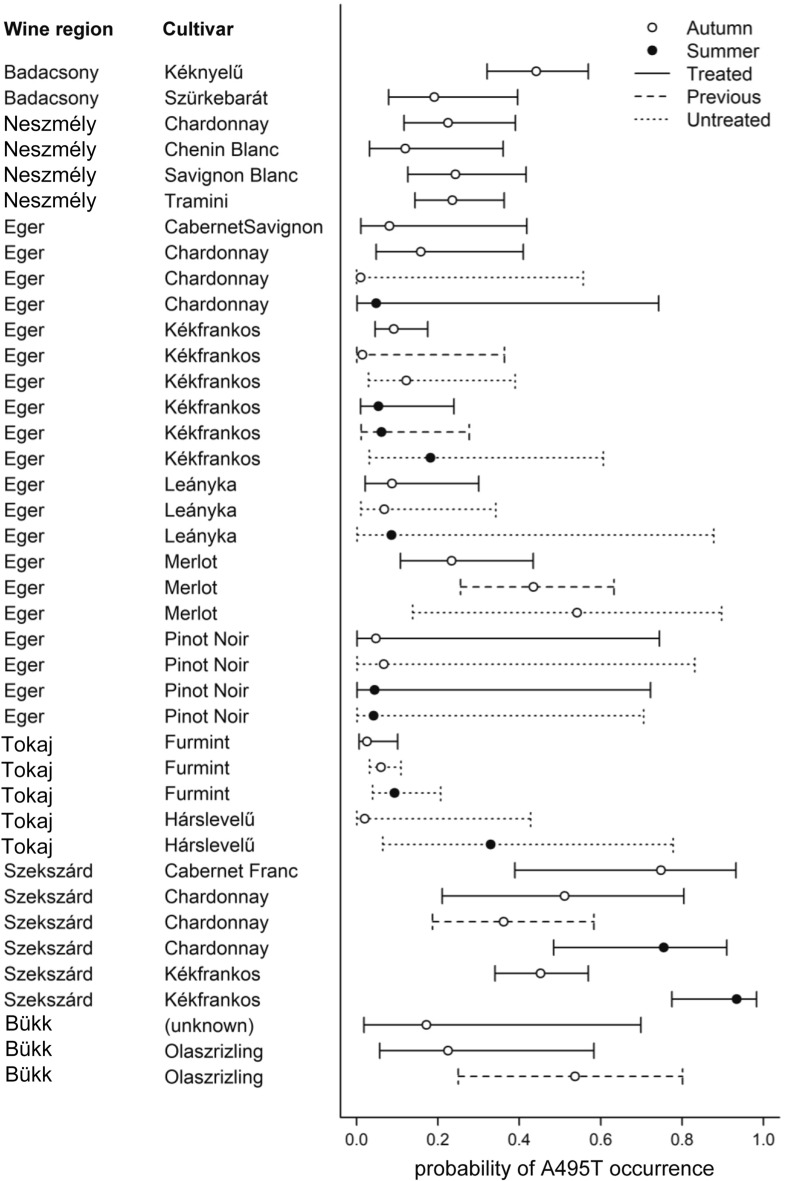
Mean probability of A495T occurrence with 84% confidence interval in each combination of vineyard, variety, and fungicide treatment in the two seasons. Non-overlapping bars indicate significant differences in the probability of A495T resistance marker occurrence.

**Figure 6:**
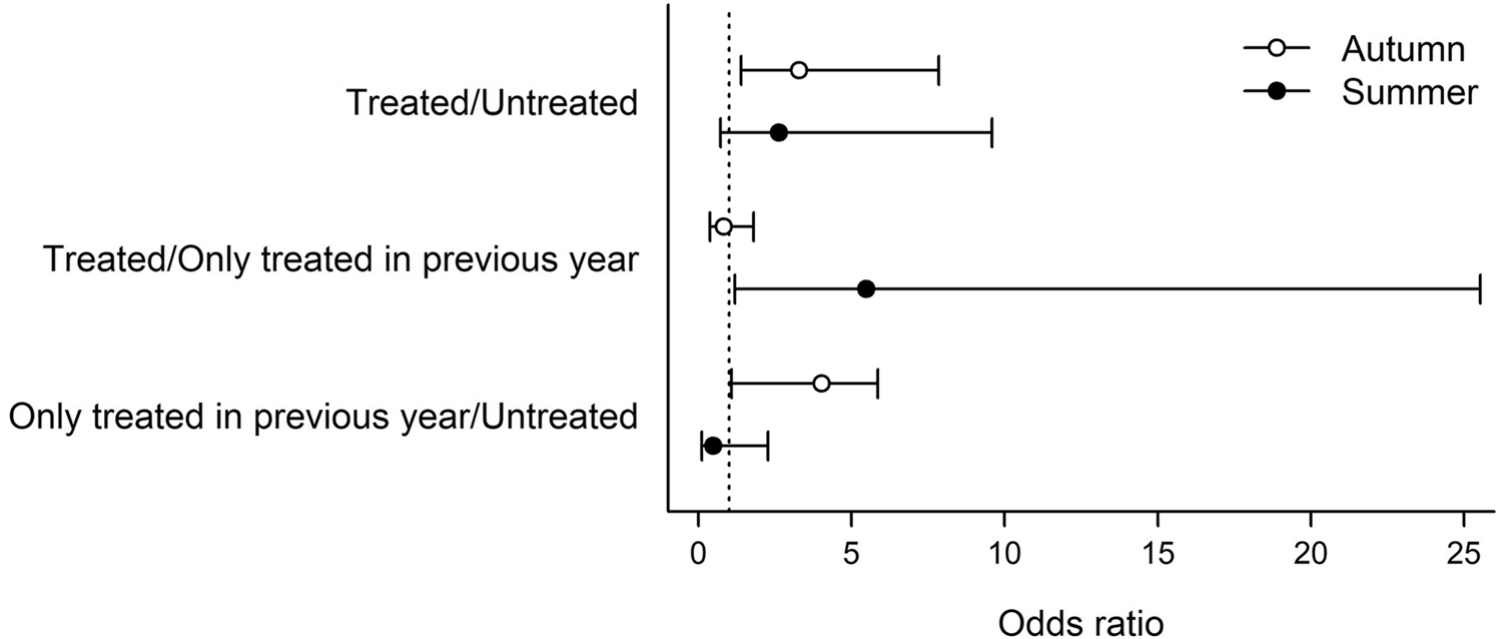
Differences between fungicide treatment groups, expressed as odds ratios (proportional difference in the odds) of A495T occurrence, averaged over all CWRCs in two seasons. Each symbol with error bar represents an odds ratio with 84% confidence interval; overlapping summer and autumn bars indicate non-significant differences in treatment effects between seasons. The vertical dotted line stands for no treatment effect (odds ratio = 1).

A495T marker of DMI resistance was detected in all wine regions, in approximately 16.8% of the assayed samples (346 of 2065; Supplementary Dataset). The proportion of samples carrying A495T varied between vineyards and between years (Fig. 4). We found significant differences in the occurrence of A495T among several combinations of wine region and cultivar (CWRC; see Methods for details), as evidenced by the lack of overlap among 84% CIs (Fig. 5). Also, the probability of A495T occurrence differed significantly between each of the three study years, whereas it did not differ significantly between *TUB2* genotypes and seasons (Table 5). The effect of fungicide treatment on the probability of A495T occurrence did not differ between summer and autumn (Fig. 6). Those CWRCs that had been treated in the given year showed a higher probability of A495T occurrence (*p* = 0.035; Table 5), but the difference between treated and untreated samples was not significant when this comparison was restricted to only those combinations from which we had both treated and untreated samples (odds ratio = 0.87, 95% CI = 0.21–3.56, *p* = 0.845). Samples that had been treated in the previous year but not in the sampling year did not differ significantly in the probability of A495T occurrence from either treated or untreated samples (Table 5).

**Table 5:** Odds ratios (proportional difference in the odds) of A495T occurrence between *TUB2* genotypes, study years, and fungicide treatments.

Variable	Contrast	Odds ratio	95% CI		*p*
			Lower	Upper	
*TUB2* genotype	C present/absent at nucleotide position 79	1.00	0.58	1.74	0.994
Season	Autumn/summer	1.03	0.42	2.49	0.952
**Year**	**2017/2018**	**5.98**	**3.07**	**11.64**	** < 0.001**
**Year**	**2017/2019**	**3.12**	**1.64**	**5.93**	**0.001**
**Year**	**2018/2019**	**0.52**	**0.34**	**0.80**	**0.003**
**Fungicide treatment**	**Treated/untreated**	**2.77**	**1.07**	**7.17**	**0.035**
Fungicide treatment	Treated/untreated—restricted*	0.87	0.21	3.56	0.845
Fungicide treatment	Treated/only treated in previous year	1.21	0.46	3.19	0.700
Fungicide treatment	Only treated in previous year/untreated	2.29	0.67	7.85	0.187

Significant differences (i.e., 95% confidence intervals that exclude 1) are highlighted in bold.

*Comparison restricted to only those combinations from which we had both treated and untreated samples.

Nucleotide positions 1119 and 1170 of *CYP51*, showed no variation in our samples (i.e., nucleotide A was found in position 1119 and G in position 1170; data not shown), except for a single isolate, which had a G-to-A change in position 1170 (Table 3).

### Co-infection and the occurrence of A495T on single leaves

Among the 321 leaves analyzed, 270 (~ 84%) carried more than two genotypes. The minimum number of genotypes present on a single leaf varied generally between 2 and 6. The highest number of genotypes found on one leaf sample was 10 (sample EL31C; Supplementary Table 2).

From the 590 additionally sampled chasmothecia, 19% carried the DMI resistance marker A495T, which is about the same value as in the total dataset (Dataset A). The proportion of chasmothecia originating from the same leaf and carrying the marker varied from 0 to 87.5% (Supplementary Table 2). Supplementary Fig. 1 shows the distribution of different genotypes on a single leaf (sample EL48B).

## Discussion

Based on our large-scale sampling of *E. necator* populations in Hungary we inferred their genetic diversity, the abundance of a fungicide resistance marker and the co-infection on single leaves. We proved the presence of 14 genotypes, among them eight new, yet unknown genotypes.

Genotype H1, which is identical to genotype 41 sensu Brewer and Milgroom^18^, was also found in Israel and was denoted as Israeli B^19^. Genotype B-NZ1 originating from New Zealand also shares identical nucleotides, however, some characteristic nucleotides were not studied when it was identified^14^, so we cannot verify if it was identical to genotype H1. Genotype H2 is most similar to another genotype B-NZ2 from New Zealand^14^, nevertheless it is also unclear if these two represent the same genotype. Genotype H3 is identical to the genotype 43, and H4 is identical to genotype 44^18^. H1/genotype 41 and H3/genotype 43 were also isolated in the Western US^18^. H4/genotype 44 was found in two European isolates^18^ and it was also frequently detected in our comprehensive sampling. H3 and H4 are similar to genotype B2 from Hungary^17^, but, as *EF1-α* sequences were not analyzed in that study, the unambiguous identity could not be determined. Genotype H7 from Hungary is identical to genotype B-IN1, reported from India^24^. Genotype H13, which is identical to genotype 11^18^ found in isolates from the United States^18^, was detected once. We did not detect samples belonging to genotype 33^18^, A-IN1^24^, EU-A and Israeli A^19^ (all corresponding to the same genotype previously known to characterize group A; Table 1).

In general, majority of the *E. necator* isolates collected outside of North America can be sorted into two well-established groups according to several SNPs of four loci^18, 19^. Diagnostic nucleotide positions in ITS, IGS, *TUB2* or *EF1-α* seemed to be equally good attributes to unequivocally differentiate genetic groups A and B in most of the earlier studies. However, the genotype Israeli B2^19^ does not fit this model, since it bears the SNPs characteristic for group B in ITS, IGS, and *EF1-α,* and has the SNP characteristic of group A in *TUB2*. Similarly, B-IN3, detected in India, is similar to group A isolates based on the ITS region, and to group B in its *TUB2* sequence^24^. Furthermore, its IGS sequence includes nucleotides characteristic to both group A and B^24^. The same genotype was reported from Israel as a new genotype, denoted as IL, and without comparing it to the genotypes from India^19^. B-IN3/IL represents another independent example for the discrepancy in grouping *E. necator* into solely two groups. Out of the fourteen genotypes identified in our work, only five (H1-H4 and H12) could be unequivocally categorized as group B genotype. Out of the remaining nine genotypes we found, seven can be rated as group B genotypes according to SNPs in ITS and IGS (H5-H11), five according to SNPs in *TUB2* (H5, H6 and H9-H11), and eight according to SNPs in *EF1-α* (H5, H6 and H10-H14). However, according to the SNPs in *CYP51,* all but one genotypes belong to group B. Based on this, our data strengthen the recommendation^24^ that discrimination of *E. necator* genetic groups should be based on several genes.

As some genotypes contain SNPs characteristics to both groups A and B, we suppose that some of our newly described genotypes may be recombinants. These either result from local sexual crosses or parasexual recombination^41^. Alternatively, or additionally, some of the genotypes present in Hungary may be introduced to the country from unknown provenances.

Although sexual recombination was found to be less common in group A in some *E. necator* populations^21, 29, 31, 40^, we frequently (~ 20%) detected genotypes belonging to group A based on position 79 of *TUB2* in chasmothecia, the sexual fruiting bodies. Neither group A nor B was detected more frequently in chasmothecia than in mycelial samples, suggesting lack of correlation between sexual reproduction and A and B genetic groups. The fact that we did not detect linkage disequilibrium in our dataset means that sexual reproduction of *E. necator* takes place regularly. Our data indicate that group A is also reproducing sexually, similarly to other results^16, 25, 32^.

Based on our data on the presence of group A genotypes in chasmothecia, the revealed diversity in Hungary, and the new genotypes, some of which we consider recombinants, we deduce that *E. necator* groups A and B mate and reproduce sexually. We assumed this previously^16^ based on a limited number of field samples, considering that the two groups were found to be interfertile, as found in laboratory experiments^33^.

The revealed genetic diversity of *E. necator* in Hungary is exceptionally high in comparison to other regions of the world, surpassed only by the diversity in the USA, the proposed source of origin of *E. necator*^18, 42^. It is possible that the previously unknown haplotypes detected from Hungary during the current study simply have not been detected in the USA. However, genetic differentiation between samples from Hungary and USA could be detected. The high number of genotypes detected in Hungary but not elsewhere, and because of the genetic differentiation between populations in Hungary and the USA, our results might support the hypothesis of a possible non-North American (presumably Asian) origin of some *E. necator* genotypes, following the reasoning of Gur et al.^19^.

We identified genetic structure in the Hungarian populations of *E. necator* among wine regions separated by a few hundred kilometers, and the genetic distance was higher with geographical distance. In addition, population structure analysis also showed genetic composition characteristic to the sampled wine regions. These results point to the isolation by geographical distance. However, no genetic differences based on geography were detected in Israel in *E. necator* populations located within a few hundred kilometers from each other^19^. On the other hand, results obtained on a smaller sampling scale (approximately 30–100 m) in North American vineyards^43^ were similar to our findings.

We found significant, although low genetic differentiation between cultivars, Kékfrankos and Chardonnay. Similarly, group IL was predominant on the wild and traditional vines, and on the table grapes, while group B was dominant on wine grapes in Israel, showing significant genetic differentiation according to host types^19^. Furthermore, we detected higher differentiation between sampling seasons than between cultivars, similar to what was detected in Israel^19^. Thus, even if the temporal isolation hypothesis^18, 21, 33^ was refuted by more recent samplings^16, 17^, our data indicate that the genetic composition of *E. necator* populations of Hungarian wine regions mainly depends on the growing season, and to a lesser extent also on grapevine variety.

Mutation A1119C was detected neither in conidial isolates, nor in field samples (data not shown), therefore, the study focused on the A495T marker. We investigated if the occurrence of A495T mutation is associated with group B. In a small scale study *E. necator* isolates belonging to group A were shown to be more sensitive to triadimenol, a DMI-type fungicide^34^. Thus, if resistance in those experiments was conferred by A495T, group B should possess the resistance marker more frequently. Contrarily, we did not detect significant difference in the occurrence of A495T between groups A and B. However, different DMI fungicides do not show complete cross-resistance^15, 38^. In addition, the A495T mutation is not the solely possible mechanism of the DMI resistance, there are alternative mechanisms^15, 23, 34, 36^, which could also explain the seemingly contrasting results.

Fungicide treatments impose selection pressure on the populations, in which the resistance levels may increase^44^. In our study we found no significant effect of the fungicide treatments on the frequency of A495T resistance marker. This apparent contradiction may be explained by the use of fungicide mixes by growers, potentially lowering the selection pressure on the resistance to DMI-type fungicides^44, 45^. Alternatively, or additionally, other resistance mechanisms of DMI resistance could explain the apparent lack of treatment effect on A495T marker frequency.

As *E. necator* is airborne, the spread of resistant genotypes containing A495T from the surrounding areas can explain the presence of the marker in untreated plots, as the migration of such genotypes into sensitive populations is possible^46^. For example, some strains *E. necator*^47^ and those of *Podosphaera xanthii*^48^*,* the fungus causing cucurbit PM, collected from untreated plants were shown to be resistant. Alternatively, if fungicide treatments were halted not long before our samplings, the strains carrying the marker can also be the descendants of such strains from earlier years.

CWRC, however, did influence the presence of A495T. This finding is in line with the results of the population genetic analyses on grapevine variety and geographic distance effecting genetic composition of the pathogen populations. Similarly, regional differences of the marker frequencies of resistance to DMI and to quinone outside inhibitors of *E. necator* in France^49^, and to succinate dehydrogenase inhibitors in the USA^50^ were also proven. These patterns likely reflect the local differences in disease pressures due to geographic location and climate^51^.

As *E. necator* has a bipolar-heterothallic mating system^20^, the presence of chasmothecia is in itself a proof for the co-infection by two different genotypes of the pathogen on the sample. In this respect, most of our samples were co-infected. This result, however, is not specific to chasmothecial sample type, as when only mycelia of *E. necator* were sampled, a similarly high level of mixed infection was detected^17^.

During our survey we found that most single-leaf samples were co-infected by at least three, and up to ten different GPM genotypes. Remarkably, in some cases, the A495T marker was present in some, but not in all chasmothecia originating from the same leaf. As the presence of A495T marker together with the wild type allele in the sample can be caused by the presence of both alleles in a single isolate^15^, as well as by co-infection, *CYP51* sequences we not considered in the co-infection analysis. Because of this strict assumption, the number of co-infecting genotypes is possibly underestimated. Altogether, co-infection by *E. necator* is common, as it is in *Podosphaera plantaginis* populations causing PM on *Plantago lanceolata*^52^ and in *E. alphitoides* infecting oak trees^53^.

A phenotyping study revealed that more aggressive strains of *P. plantaginis* form co-infections more likely than less aggressive strains^54^. The aggressiveness of the detected *E. necator* genotypes is unknown, but we assume that the more widespread genotypes or strains may be more aggressive and/or more successful in spreading and colonization, as it was found for certain *E. necator* genotypes^19, 26, 33, 39^. However, genotypes characterized by differences in their aggressivity may be maintained by co-infection^5^. We cannot exclude that the high-level of co-infection in *E. necator* populations helps in maintaining genetic diversity of the pathogen.

## Conclusion

Taking together our data and recent results^16, 17^ we conclude that several genotypes of *E. necator* can be differentiated in Europe, rather than solely groups A and B. Thus, the binary genetic group concept should be abandoned. The fundamental role of the locality in the genetic structure of *E. necator* populations and on the distribution of the resistance-associated marker stress the importance of the local monitoring to understand the evolution and spread of this important pathogen.

## Materials and methods

### Sampling

GPM samples were collected from 2017 to 2019 in six wine regions (Eger, Tokaj, Szekszárd, Badacsony, Bükk, and Neszmély) of Hungary, located approximately 70–320 km from each other. Sampling sites, 15 vineyards altogether, were chosen to represent different grapevine cultivars, training systems, and conventional and organic farming (Supplementary Dataset). Samples were taken twice a year, in early summer (May–July) and at the end of the season (September- November). In Badacsony, Bükk, and Neszmély, samples were collected only at the end of the season due to the lack of GPM in early summer. Depending on the shape and size of the vineyards six to eight plants were sampled from three or four rows during each sampling (18–32 plants/vineyard). In early summer, infected leaves and grape berries, while at the end of the season only infected leaves were collected. Samples were placed in paper bags and transported to the laboratory. In total, more than 1500 GPM field samples were obtained. Additionally, samples were taken in Budapest in two years, to include samples from a direct producer hybrid grapevine. These samples were used only for initiating in vitro isolates.

All actions, including sampling and experiments during this study complied with all institutional, national, and international guidelines and legislation. Sampling was always done with the knowledge, consent and permission of the owners and/or growers of vineyards.

### In vitro plant material and isolation of *E. necator*

Plant tissue culture technique was used to produce in vitro grape plants with aseptic and susceptible grape leaves for isolation of *E. necator* strains. Young shoots were grown from grape cuttings (cv. ‘Chardonnay’ and ‘Kékfrankos’) in the laboratory under daily illumination. Grapevine stems, 5–10 cm long, were cut and surface sterilized in 1% calcium hypochlorite for 15 min, rinsed three times with sterile deionized water, and air-dried in a sterile laminar flow hood. Then stems were placed into plastic containers with Murashige and Skoog (MS) medium (Murashige & Skoog medium Mod. No. 1B, Duchefa Biochemie), solidified with 6.5 g/l Phyto agar (Duchefa Biochemie); supplemented with 500 µg/l (2.46 µM) indol-3-butyric acid (IBA; Duchefa Biochemie) to enhance the formation of roots. After two months, leaves, at least two cm diameter, were cut from the in vitro plantlets and placed in Petri dishes containing MS media without IBA. Plantlet stems were cut into two or three node fragments and were placed in new containers for a continuous supply of plant material.

*Erysiphe necator* was isolated from field-collected PM colonies. To obtain isolates, and later, during the passages, a single PM conidium or conidial pseudochain was picked using a glass needle under a dissecting microscope in a laminar flow hood, and placed on the upper surface of an in vitro grapevine leaf in a Petri dish. Inoculated leaves were incubated for 14 days at 22 °C under 12-h/day illumination. Colonies were treated as single-conidial isolates after two passages. We obtained 59 isolates (Table 3).

### DNA extraction and genotyping

Chasmothecia are considered the smallest discrete units of PM fungi that can be easily handled, and provide sufficient amount of DNA for molecular biology analyses^16^. Therefore, from samples collected in autumn, chasmothecia were collected and DNA was extracted from single chasmothecia (one chasmothecium/leaf sample) as described previously^16^. For DNA extraction from samples without chasmothecia (field samples collected in summer, and from in vitro isolates), fungal material was collected by touching an ~ 1 cm^2^ piece of office cellotape (Henkel Pritt) to the surface of the infected leaves and berries. The tape was boiled in 100 µl of TE buffer for 10 min in a 1.5 ml centrifuge tube, then 1 μl of the solution was used as the target in PCR amplifications.

To study the possible co-occurrence of different genotypes on single leaves, additional chasmothecia were collected from distinct regions of the leaf surfaces. These 321 leaves were selected based on carrying abundant chasmothecia widespread on the leaf surface. From these leaves, additional chasmothecia were sampled and used for DNA extraction, in addition to the single chasmothecium sampled first (see above). From 266 leaves, one additional; from 25 leaves, two; and from eight leaves, 3–5 additional chasmothecia were sampled, and from 22 leaves, almost fully covered with powdery mildew, seven or more additional chasmothecia were collected. This sampling resulted in altogether 590 further single chasmothecial DNA samples.

Multiple loci were amplified and sequenced from the extracted DNA. PCR-amplifications and sequencing of ITS, IGS and *TUB2* were carried out as described previously^16^. EF1-6^18^ and EF1-5alt (GATCGCAACAATGAGCTGCTT) primers were used for PCR-amplification and sequencing for *EF1-α*. EF1-5alt was designed by aligning the sequence of EF1-5^18^ to *EF1-α* reference sequences downloaded from whole genome sequence data^23^ using MEGA7^55^, and adjusting the primer sequence to avoid potential mismatches. *TUB2* and *EF1-α* loci were PCR-amplified and sequenced from 1963 and 1835 samples, respectively. ITS and IGS regions were sequenced from a subset of samples (580 and 557, respectively) as these loci showed low variability (see below). For detection of the A495T nucleotide substitution in the *CYP51* gene, direct sequencing of the 5’ region of the gene encompassing position 495 was applied for non-chasmothecial samples, and real-time PCR assay was used for genotyping chasmothecial samples; both as described^16^. The occurrence of the A495T marker was investigated in 2065 samples in total. The 3’ region of *CYP51* was sequenced from 138 single chasmothecial samples and all 59 isolates (a total of 197 sequences) using the same PCR protocol^16^ with primers EnCYP1055F and EnCYP1752R^36^.

Electrophoregrams were processed and individually checked using the CodonCode Aligner 8.0.2 (CodonCode Corporation, USA). The four sequenced loci^18, 19^ were checked for SNPs. Possible variations of *CYP51* were also investigated, especially at positions 143, 608, 1170 and 1620, as these sites are characteristic to the genetic groups A and B^21^, and positions 495 and 1119, which were reported as markers of DMI resistance^15, 36^. GenBank accessions GQ255473 (ITS), GQ255476 (IGS), GQ255475 (*TUB2*), GQ255471 (*EF1-α*), and U83840 (*CYP51*) were used as references. Nucleotide positions showing double peaks on chromatograms were considered to contain both alleles in the DNA samples^17, 56, 57^. Representative sequences obtained in the present study were deposited in NCBI GenBank under accession numbers OQ709801-OQ709802, OQ709882 and OQ723652-OQ723677.

### Datasets

The real-time PCR assay resulted in presence-absence data. *CYP51* sequence results were manually converted into presence-absence data, based on the nucleotide position 495 of *CYP51* on the chromatograms. The final complete dataset (Dataset A; Supplementary Dataset) contained both variable nucleotide data and A495T marker presence-absence data from 2148 field samples, originating from 1558 individual grapevine leaves or berries. Dataset A was filtered for the subsequent analyses with Microsoft Office Excel 2013. To define genotypes, we used a dataset (Dataset B) of 521 samples, from which all four loci were determined. These included 462 field samples, and all of the 59 in vitro isolates (Table 3). SNP data of samples with one double peak detected on chromatograms were separated into two rows in the dataset, hence resolving the ambiguity caused by the two different nucleotides present at the variable position. This process resulted in two rows from one sample, each representing one of the two genotypes present in the DNA extract. These chasmothecia were considered to contain both of those genotypes when calculating the number of samples belonging to each genotype. To prevent sample-size bias, samples not showing any ambiguous positions were duplicated in the dataset before calculating genotype numbers and frequencies, as *E. necator* is a heterothallic fungus^58^, and therefore, two different isolates (“individuals”) are needed for chasmothecia to form, even if they show no differences in the sequenced loci. Thus, altogether 983 entries (including 924 data from 462 field samples and 59 from isolates (single entries each)) were considered for defining genotypes and for assigning samples to the defined genotypes. Sequence data from isolates were only included in Dataset B.

A third dataset with 2108 entries from 1054 samples (Dataset C), which contained field samples of which *TUB2* and *EF1-α* could be fully sequenced (irrespective of ITS and IGS), and in which ambiguous nucleotide positions were also treated as above, was used for STRUCTURE analysis (see below) and for visualizing the geographical and temporal distribution of samples.

To create the dataset for other population genetic analyses (Dataset D), Bükk wine region, represented with small sample size, from only one small vineyard from unknown grapevine varieties, was excluded. Samples without any *TUB* and *TEF* sequence data were also omitted. *CYP51* was also omitted as it is under selection pressure by fungicide treatments^23^. In the samples where one ambiguous nucleotide position was found, genotype was resolved as above. Data of samples not showing any ambiguous positions were duplicated, as above. The final Dataset D contained 1694 samples (3388 entries).

Data from leaves with more than one chasmothecium sampled, were used to analyze the co-infection level on single leaves, by comparing sequences obtained from different chasmothecia from the same leaves. For this, samples representing double peaks in more than one SNP positions were also included to calculate the minimal number of genotypes present on single leaves. In these analyses, *CYP51* was omitted as it is known that it may be present with more alleles in a single genome^15, 23^.

### Multilocus haplotype network

To infer and visualize the genetic similarities of the genotypes detected, a multilocus haplotype network was created using 37 variable nucleotide positions of ITS, IGS, *TUB2* and *EF1-α*. Network was reconstructed with PopART v. 1.7^59^ using the TCS method^60^. In the first analysis genotype frequency data from Hungary were included. In a second analysis, presence-absence data in Hungary and other locations of the world (other European countries, USA, Israel, India and Australia)^18, 19, 24^ were included.

### Linkage disequilibrium and genetic diversity

To assess deviations from random mating in the whole sampled population, Multilocus v1.3b^61^ was used. Association indexes (I_A_, and $\overline{r }$_d_, an index independent from the number of studied loci) were estimated from the complete dataset. Genotypic diversity (a measure of the probability that two randomly selected samples are of different genotype), the number of different genotypes detected, and the frequency of the most frequent genotype were also calculated. “Fix missing data during randomizations” option of the software was in effect. To test statistical significance, 1000 random permutations were run and actual data were compared to the randomized dataset to determine *p-*values.

As population structure was identified in the dataset (see below), the detected populations were also characterized separately. For this, five individual populations were introduced and population indexes were calculated using Multilocus v1.3b for each population as above.

### Population structure analyzes

The Bayesian clustering program STRUCTURE 2.3.4^62^ was used to determine population structure. Five independent analyzes were carried out, from K (number of clusters) = 2–7, with admixture models and 250,000 Markov Chain-Monte Carlo (MCMC) iterations, after a burn-in of 100,000 steps. Population structure was then displayed graphically with DISTRUCT v1.1^63^. We used the Evanno method^64^, via the STRUCTURE Harvester website (http://taylor0.biology.ucla.edu/structureHarvester/)^65^, to identify the K value corresponding to the most supported structure.

To analyze the partition of genetic variation within and among populations, we used Analysis of Molecular Variance (AMOVA)^66^ with GenAlEx v6.5^67^. PhiPT (ФPT) value, the measure of genetic differentiation was determined (i) among distinct subpopulations shown by STRUCTURE, (ii) among samples collected in three sampling years, and (iii) between samples collected in two sampling seasons, late spring–summer, and autumn. We also analyzed (iv) the possible effects of grapevine cultivar on the genetic composition. For this, data from samples from cultivars Kékfrankos (n = 906) and Chardonnay (n = 180), originating from Eger and Szekszárd were involved; these two cultivars were thoroughly sampled in these two regions. The sample sizes in this analysis were: 84 for Chardonnay samples originating from Eger and 96 from Szekszárd; 488 for Kékfrankos samples from Eger and 418 from Szekszárd.

Significant genetic differentiation was determined using comparisons to 999 random permutations of data with GenAlEx v6.5. Distance Calculation was set to “Haploid”; missing data were coded as zeros and “Interpolate Missing” function was in effect, except for analysis (iii) where ITS sequence data were insufficient for interpolation. Other options were as defaults.

To test correlation between genetic and physical distances of the collected *E. necator* samples, Mantel test was applied^43^ on a geographical distance (in kilometers) matrix compiled from Google Maps data and a genetic similarity matrix. The latter was created using MEGA7^55^ by calculating mean pairwise nucleotide differences between populations in each combination. For gaps and missing data, pairwise deletion was in effect. The observed data were compared to 999 randomly permuted datasets for frequency distribution and determination of *p*-value in the Mantel test.

### Sexual reproduction within genetic groups A and B

To infer if groups A and B significantly differ in the frequency of forming chasmothecia, a Chi-squared test was conducted with the data on the sample belonging to group A or B, based on *TUB2* (i.e. with SNP in position 79 of *TUB2*; n = 1410) and the sample type (chasmothecium or mycelium). The calculation was conducted in R v4.0.3^68^.

### Analyzes on A495T fungicide resistance marker data

A495T marker presence-absence data were used to analyze the effects of (i) wine region, cultivar and DMI fungicide treatment, (ii) season and (iii) year of collection on the probability of the presence of A495T mutation. Furthermore, (iv) the possible correlation of SNP present at nucleotide position 79 of *TUB2*, traditionally associated with genetic groups A and B, with the presence of A495T marker was investigated. For this, all samples diagnosed positive with the real-time PCR method, and all samples found to carry the A495T mutation based on sequencing were included. Treatment history was recorded as a three-category factor: untreated; treated in the previous year(s) and in the year of sampling; and treated in the previous year(s), but not in the year of sampling.

We analyzed the probability of A495T occurrence with a generalized linear model with binomial error and logit link, using independent samples (data from only one chasmothecium per leaf; n = 1410). Because fungicide treatment was not statistically independent of the wine region and cultivar (χ^2^ tests: *p* < 0.001), we combined these three variables (wine region, cultivar, and fungicide treatment) into a single categorical factor to avoid multi-collinearity among the explanatory variables in the model. We included this combined categorical factor, season (summer or autumn), the two-way interaction of the latter two variables, *TUB2* genotype (presence/absence of group B coded as binary variable based on position 79, i.e. nucleotide C present or absent), and year as explanatory variables (fixed factors). In the model we did not include sample type (i.e. whether the sample originated from mycelia or chasmothecia), because including it would have resulted in multi-collinearity (variance inflation factor > 9). However, it is unlikely that ignoring sample type would have biased the results, because the relative frequency of *TUB2* genotypes was similar between the two sample types (see Results). To handle separation in the binomial model (i.e. lack of variance in A495T occurrence in certain explanatory categories), we ran the analysis using the median-bias reducing score adjustments^69^, as implemented in the brglm2 package of R v4.0.3^68^. To test the effects of the explanatory variables, we calculated linear contrasts from the model’s estimates, using the emmeans package of R, as follows. First, we tested whether the effect of treatment differed between seasons. To this end, we estimated the treatment effects (i.e. pairwise differences among the three treatment categories) separately for each season, and we compared the 84% confidence interval (CI) of each treatment effect between summer and autumn. The lack of overlap between two 84% CIs indicates a significant difference, i.e. that the 95% CI of the difference does not include zero^70^. Second, we tested the effects of treatment (regardless of season) in two ways. In the first approach, we compared the average occurrence of resistance among the three treatment categories as described above, but not separating the data by season. In the second approach, we restricted the same calculation to only those combinations of wine region and cultivar (henceforth, CWRC) for which we had data from both treated and untreated samples. Finally, we estimated the differences in A495T occurrence between *TUB2* genotypes and among years by pairwise linear contrasts, and we assessed the differences in A495T occurrence between CWRCs by the overlap of their 84% CIs.

### Supplementary Information

Supplementary Information 1.

Supplementary Information 2.

## Data Availability

All relevant data, except for sequences, are included in the manuscript and its supplementary files. Representative sequences were deposited in NCBI Genbank (https://www.ncbi.nlm.nih.gov/genbank/), under accession numbers OQ709801-OQ709802, OQ709882 and OQ723652-OQ723677, and are freely available.
