# Supplementary material for: Comprehensive analyses of the occurrence of a fungicide resistance marker and the genetic structure in Erysiphe necator populations
Source: Sci Rep. 2023 Sep 13;13:15172. doi: 10.1038/s41598-023-41454-1 (PMC10499922; doi:10.1038/s41598-023-41454-1)
Supplement: Supplementary file 2 — Supplementary Information 2. [file 41598_2023_41454_MOESM2_ESM.doc]

**Comprehensive analyses of the occurrence of a fungicide resistance marker and the genetic structure in *Erysiphe necator* populations**

Alexandra Pintye^1,a^, Márk Z. Németh^1,a,*^, Orsolya Molnár^1,a^, Áron N. Horváth^1^, Fruzsina Matolcsi^1,2^, Veronika Bókony^1^, Zsolt Spitzmüller^3^, Xénia Pálfi^3^, Kálmán Z. Váczy^3^, Gábor M. Kovács^1,2^

^a^: AP, MZN, and OM contributed equally to this work

*: corresponding author (e-mail: nemeth.mark@atk.hu)

^1^ Plant Protection Institute, Centre for Agricultural Research, ELKH, Budapest, Hungary

^2^ Department of Plant Anatomy, Institute of Biology, Eötvös Loránd University, Budapest, Hungary

^3^ Food and Wine Research Institute, Eszterházy Károly Catholic University, Eger, Hungary

**SUPPLEMENTARY INFORMATION**

Supplementary information of this manuscript consists of a Supplementary Dataset, two Supplementary Tables and one Supplementary Figure.

**Supplementary Dataset**

Dataset A: list of samples, details on sample origins, and results of multilocus sequencing and qPCR-based genotyping. The dataset was uploaded as a separate file. See “*Supplementary Dataset - Dataset A.xls*”.

**Supplementary Table 1**

**Results of population genetic analyses conducted with Multilocus v1.3b.**

| **Wine region** |  | ***NumDiff*^a^** | ***MaxFreq*^b^** | ***Diversity*** | ***PrCompat*^c^** | ***IndAssoc* ^d^** | ***rBarD* ^e^** |
| --- | --- | --- | --- | --- | --- | --- | --- |
| **All wine regions** | observed | 12 | 1089 | **0.681** | 0.667 | -0.326255 | -0.124067 |
|  | *p* value | 0.748 | 0.816 | 0.707 | 0.324 | 0.69 | 0.69 |
| **Badacsony** | observed | 10 | 93 | **0.683** | 0.667 | 0.0615244 | 0.0210938 |
|  | *p* value | 0.464 | 0.951 | 0.264 | 0.789 | 0.327 | 0.327 |
| **Neszmély** | observed | 7 | 264 | **0.565** | 0.733 | -0.0300657 | -0.0107595 |
|  | *p* value | 0.273 | 0.523 | 0.721 | 0.859 | 0.776 | 0.776 |
| **Eger** | observed | 11 | 370 | **0.653** | 0.667 | -0.289759 | -0.107212 |
|  | *p* value | 0.789 | 0.774 | 0.816 | 0.616 | 0.791 | 0.791 |
| **Tokaj** | observed | 11 | 359 | **0.672** | 0.667 | -0.342394 | -0.154972 |
|  | *p* value | 0.999 | 0.893 | 0.58 | 1 | 0.179 | 0.179 |
| **Szekszárd** | observed | 5 | 321 | **0.623** | 0.800 | -0.457173 | -0.295787 |
|  | *p* value | 0.395 | 0.911 | 0.058 | 1 | 0.08 | 0.08 |

^a^ Number of different genotypes

^b^ Frequency of the most frequent genotype

^c^ Probability of two individuals sampled randomly having different genotypes

^d^ Traditional measure of multilocus linkage disequilibrium

^e^ Standardized measure of multilocus linkage disequilibrium

**Supplementary Table 2**

**List of leaf samples from which at least eight chasmothecia were collected.** The number of chasmothecia collected from single leaves, the minimal number of possible haplotypes (calculated based on the number of ambiguous SNP positions), and the number and ratio of chasmothecia carrying the A495T mutation are given.

| **Sample designation** | **Number of sampled chasmothecia** | **Minimal number of haplotypes present** | **Number of chasmothecia carrying the A495T mutation** | **Portion of chasmothecia carrying the A495T mutation** |
| --- | --- | --- | --- | --- |
| E2K7A | 8 | 4 | 0 | 0% |
| E3K23B* | 8 | 1 | 1 | 12.5% |
| E3S14B | 8 | 4 | 1 | 12.5% |
| E3S3B | 14 | 4 | 3 | 21.4% |
| E3S4B | 8 | 2 | 0 | 0% |
| EC32B* | 12 | 1 | 1 | 0.8% |
| EC46B | 8 | 5 | 0 | 0% |
| EK36B | 8 | 5 | 0 | 0% |
| EL31C | 35 | 10 | 9 | 25.7% |
| EL42B | 8 | 6 | 0 | 0% |
| EL48B | 8 | 4 | 1 | 12.5% |
| M1F16B* | 8 | 1 | 0 | 0% |
| M1F1A | 8 | 4 | 0 | 0% |
| M2F16B | 8 | 3 | 0 | 0% |
| M3F25B | 15 | 6 | 0 | 0% |
| M3F8B | 24 | 6 | 1 | 4% |
| N1C27B | 8 | 4 | 7 | 87.5% |
| N1T26C | 8 | 4 | 7 | 87.5% |
| N3T32B | 8 | 4 | 6 | 75% |
| N3T9B | 31 | 6 | 24 | 77.4% |
| SO4B | 8 | 4 | 4 | 50% |
| SO6B | 16 | 4 | 1 | 6% |

* Only ITS and IGS were sequenced from these samples.

**Supplementary Figure 1**


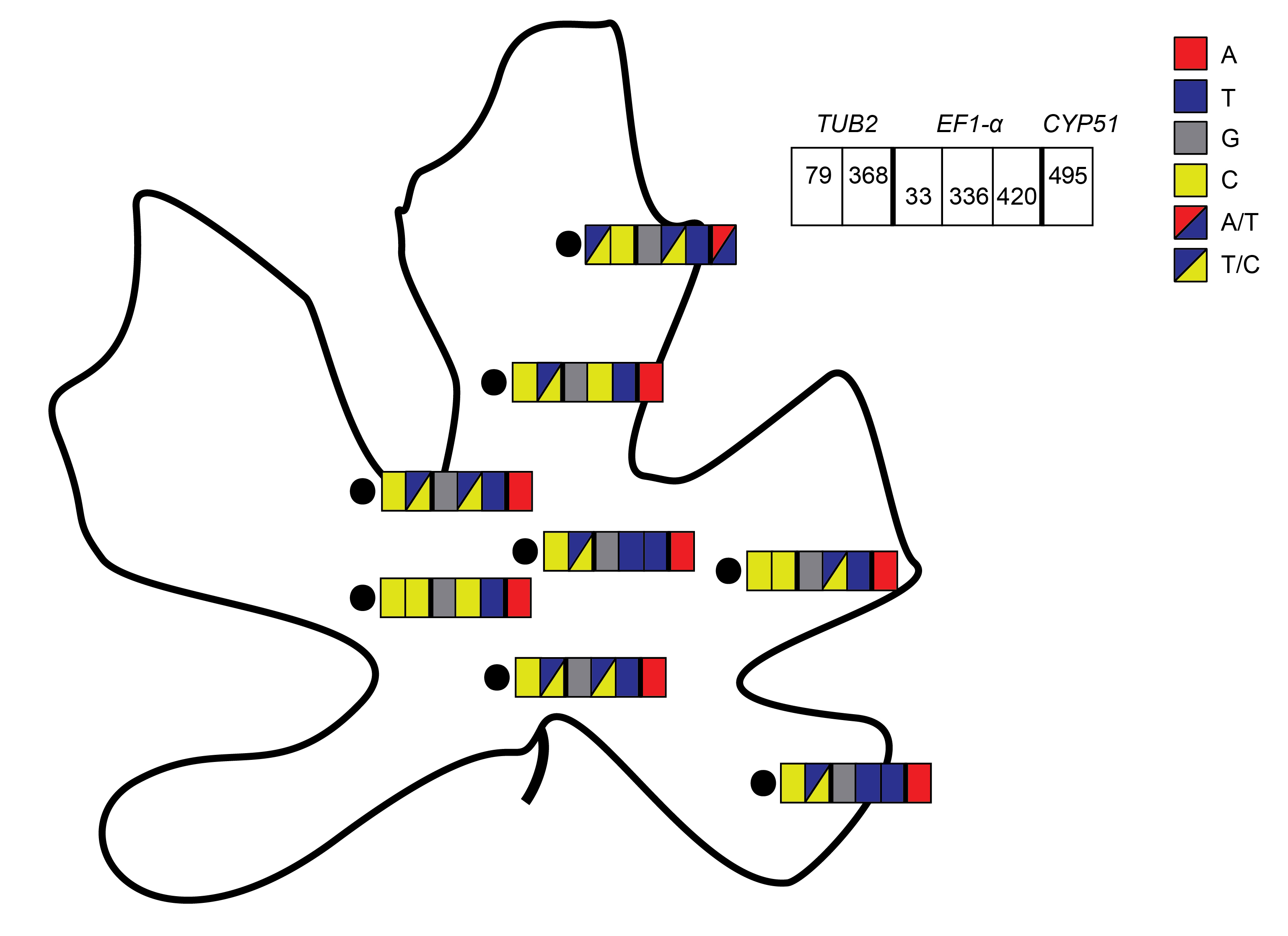


**Supplementary Figure 1. Distribution of genotypes and the presence of the fungicide resistance marker A495T at the leaf level.** The position of the black dots on the leaf shows the chasmothecia from which the DNA has been extracted. The numbers in the rectangles show the positions of the variable SNPs in *TUB2*, *EF1-α*, and *CYP51*. The leaf (EL48B) was collected in the Eger wine region in autumn 2017.
